# Advanced bioinformatics rapidly identifies existing therapeutics for patients with coronavirus disease-2019 (COVID-19)

**DOI:** 10.1186/s12967-020-02430-9

**Published:** 2020-06-25

**Authors:** Jason Kim, Jenny Zhang, Yoonjeong Cha, Sarah Kolitz, Jason Funt, Renan Escalante Chong, Scott Barrett, Rebecca Kusko, Ben Zeskind, Howard Kaufman

**Affiliations:** Immuneering Corporation, 245 Main Street, Cambridge, MA 02142 USA

**Keywords:** Artificial intelligence, Bioinformatics, Computational Biology, Coronavirus, Drug therapy

## Abstract

**Background:**

The recent global pandemic has placed a high priority on identifying drugs to prevent or lessen clinical infection of severe acute respiratory syndrome coronavirus 2 (SARS-CoV-2), caused by Coronavirus disease-2019 (COVID-19).

**Methods:**

We applied two computational approaches to identify potential therapeutics. First, we sought to identify existing FDA approved drugs that could block coronaviruses from entering cells by binding to ACE2 or TMPRSS2 using a high-throughput AI-based binding affinity prediction platform. Second, we sought to identify FDA approved drugs that could attenuate the gene expression patterns induced by coronaviruses, using our Disease Cancelling Technology (DCT) platform.

**Results:**

Top results for ACE2 binding iincluded several ACE inhibitors, a beta-lactam antibiotic, two antiviral agents (Fosamprenavir and Emricasan) and glutathione. The platform also assessed specificity for ACE2 over ACE1, important for avoiding counterregulatory effects. Further studies are needed to weigh the benefit of blocking virus entry against potential counterregulatory effects and possible protective effects of ACE2. However, the data herein suggest readily available drugs that warrant experimental evaluation to assess potential benefit. DCT was run on an animal model of SARS-CoV, and ranked compounds by their ability to induce gene expression signals that counteract disease-associated signals. Top hits included Vitamin E, ruxolitinib, and glutamine. Glutathione and its precursor glutamine were highly ranked by two independent methods, suggesting both warrant further investigation for potential benefit against SARS-CoV-2.

**Conclusions:**

While these findings are not yet ready for clinical translation, this report highlights the potential use of two bioinformatics technologies to rapidly discover existing therapeutic agents that warrant further investigation for established and emerging disease processes.

## Background

Coronaviruses are single-stranded, positive-sense, RNA viruses belonging to the *Nidovirales* order and have been divided into four broad groups (i.e., alpha, beta, gamma, delta) based initially on serology and later by phylogenetic clustering [1]. To date, seven human coronaviruses, restricted to the alpha and beta subgroups, have been identified with the first infection reported in 1967 and thought to be associated with mild, self-limited respiratory illness [2]. More recently, coronaviruses have been involved in 15–30% of upper respiratory tract infections annually with more severe clinical courses in neonates, older individuals and immunosuppressed patients. In 2002, an outbreak of severe acute respiratory syndrome (SARS) in Guangdong China was traced to SARS-CoV, a new beta-coronavirus. During this outbreak nearly 8100 patients were diagnosed with an overall mortality of 9%, which increased to 50% in patients over 60 years of age [3]. The disease was thought to have originated from infected bats and was easily contained as transmission appeared to require direct contact with infected individuals. A distinct group 2c b-coronavirus, genetically related to bat coronaviruses, was responsible for another outbreak in Saudi Arabia in 2012 and the disease was termed Middle East Respiratory Syndrome (MERS). This virus was associated with an initial 50% mortality but did not spread appreciably outside the region [4]. An outbreak of an unknown respiratory illness in Wuhan China was reported in late December of 2019 and the causative agent was identified as SARS coronavirus (SARS-CoV-2) and the disease was called coronavirus disease 2019 (COVID-19) [5]. The disease has rapidly become a global pandemic and a major priority has been placed on finding drugs that prevent or limit viral propagation and infection.

Coronaviruses share a large genome, around 30 kB, express large replicase genes encoding non-structural proteins involving approximately 20 kB of the genome, undergo early transcription of the replicase gene, contain a viral envelope, and utilize ribosomal frameshifting for non-structural gene expression [6]. The viral genome is composed of a 5′-cap structure with a leader sequence and untranslated region (UTR) composed of multiple stem loop structures needed for RNA replication [7]. The 3′-end contains an UTR that has RNA structures necessary for viral RNA synthesis as well as a 3′-poly(A) tail that mimics mRNA allowing translation of replicase-encoded non-structural proteins. Transcriptional regulatory sequences (TRSs) are found at the 5′-end of most structural and accessory genes with most accessory genes being non-essential but modulating viral pathogenesis [8]. There are four main structural proteins, termed spike (S), membrane (M), envelope (E) and nucleocapsid (N). The S protein is about 150 kD and is responsible for the “spike” on the viral surface and trimeric S protein is used for viral attachment to cell entry receptors [9].

The life cycle of human coronaviruses begins with viral attachment via the S protein to cell entry receptors, typically peptidases. The SARS-CoV virus uses the angiotensin converting enzyme 2 (ACE2) as the main cellular receptor with the membrane serine protease, TMPRSS2, acting as an accessory protein to stabilize cell entry and cleavage of the S protein following viral fusion with the cell membrane [10, 11]. The virus enters and replicates within the cytoplasm starting with translation of the replicase gene and assembly of a viral replicase complex [12]. The complex and non-structural genes act to inhibit host cell translation while promoting host mRNA degradation and enhancing viral RNA synthesis and replication [12]. The process results in genomic and subgenomic RNA generated via negative-strand intermediates and the S, E, and M structural proteins enter the endoplasmic reticulum (ER) and move into the ER-Golgi intermediate compartment where viral genomic progeny are encapsidated by the N protein [13]. After assembly virions are transported to the cell surface and released by exocytosis. In some coronaviruses excess S protein can mediate cell fusion with neighboring cells, a process that may allow rapid viral transmission without detection by the host humoral immune response [14].

To accelerate pharma R&D across targets and disease areas, Immuneering developed Disease Cancelling Technology (DCT) to identify targets and drugs reversing disease gene expression and Fluency, a computational platform for large scale high throughput in silico screening. DCT quantifies similarity of genome-wide signatures of disease to signatures of drug induced gene expression changes using cosine similarity. Uniquely relative to other methods, DCT quantifies the per-gene contribution to overall disease amplification or cancellation and is not biased to any specific targets or pathways. Fluency predicts quantitative binding affinity purely from sequence. Unlike other methods, Fluency is a single universal quantitative structure–activity relationship (QSAR) model able to accept any molecule and protein sequence as input. When trained on the over 2 million IC50 values from Chembl, Fluency achieves near experimental level binding prediction accuracy as well generating predictions on the binding site. We applied these platforms to determine if repurposing of existing drugs may be helpful in COVID-19 infection, by: (1) assessing established drugs for binding to ACE2 and TMPRSS2, two proteins used by the virus to enter cells and (2) Scanning FDA approved compounds for transcriptomic disease cancellation of coronavirus associated gene expression changes.

## Results

Given that the COVID-19 virus uses angiotensin converting enzyme 2 (ACE2) as the main cellular receptor to enter the cell, we ran two Fluency models with ACE2 as the target input. Two different Fluency models (“model a” and “model b”) were run to predict binding of ACE2 to all chemicals in the Selleckchem FDA approved drug library. Initial ranking by performance in model a is shown in Table 1, which included multiple known ACE inhibitors scoring well (Enalaprilat, Ramipril, Lisinopril, Monopril, Captopril). Out of these drugs, Enalaprilat has the best binding score from model a. Given reports of the possibility of ACE2 induction being driven by ACE1 inhibition [15] and multiple subsequent reports hinting at benefit from ACE inhibition [16–19], we were interested to observe ACE2 specificity in comparison to ACE1. For top hits, the binding of ACE2 and ACE1 was compared by calculating the difference in predicted binding (ACE2 binding minus ACE1 binding) using two Fluency models (Table 1). According to model a, Brigatinib, Tirofiban Hydrochloride, and Aleuritic Acid are top ranked by pBind, and Brigatinib is also highest ranked by model a as specific for ACE2 over ACE1. Glutathione was ranked in 7th place by model a for being more specific to ACE2 over ACE1. Next, a consensus ranking using the results of both models a and b was used to select top ACE2 binders (Table 2). Enalaprilat, Tirofiban hydrochloride, and Sotagliflozin showed balanced performance in both models. In order to assess specificity, fluency was run on top hits in reverse (predicting binding of a small molecule to the human proteome). By this metric, Ramipril, Piperacillin Sodium and Captopril had high ranking for ACE2 (Table 2). The worst score by far of top hits considered was R-406.

**Table 1:** Top ranked fluency hits for binding to ACE2, ranked by pBind in model a

Name	pBind_a_rank	pBind_a	pBind_b_rank	pBind_b	pBind_a_ACE2-pBind_a_ACE	pBind_b_ACE2-pBind_b_ACE
Brigatinib	1	8.46	978	6.44	3.99	− 0.33
Tirofiban Hydrochloride	2	8.43	123	8.27	0.64	1.17
Aleuritic Acid	3	8.06	1129	6.25	0.28	− 0.06
Enalaprilat dihydrate	4	7.90	77	8.42	0.02	0.48
Ceritinib	5	7.88	1183	6.19	3.43	0.48
Monopril	6	7.84	233	7.98	0.04	0.56
Trandolapril	7	7.73	113	8.30	− 0.03	0.54
Lisinopril	8	7.72	172	8.15	− 0.29	− 0.02
Benazepril	9	7.61	653	6.89	0.81	− 0.56
Nateglinide	10	7.59	274	7.86	0.67	2.30
Captopril	11	7.54	253	7.91	0.05	0.31
Temocapril HCl	12	7.51	661	6.88	0.61	− 0.35
Benazepril hydrochloride	13	7.51	667	6.87	0.77	− 0.56
LCZ696	14	7.22	944	6.47	0.75	0.88
Fosamprenavir calcium salt	15	7.16	1443	5.95	− 0.30	− 0.02
Thioctic acid	16	6.90	772	6.70	0.96	1.02
Zofenopril calcium	17	6.90	974	6.45	0.76	0.09
Ramipril	18	6.85	155	8.20	− 0.45	0.21
Moexipril HCl	19	6.83	1360	6.02	− 0.04	− 1.12
Perindopril Erbumine	20	6.80	190	8.10	− 0.73	0.47
Edetate Trisodium	21	6.72	1191	6.18	− 0.13	− 0.09
Enalapril maleate	22	6.72	1313	6.07	− 0.38	− 0.58
Valbenazine tosylate	23	6.66	416	7.45	− 0.28	0.36
Glutathione	24	6.65	252	7.91	1.46	0.21
Icotinib	25	6.64	928	6.49	2.22	− 0.30

For each version of fluency run (models a and b), the predicted binding and rank is reported. A higher “pBind” signifies a higher binding affinity. The difference in pBind between ACE2 and ACE is reported in the last two columns, with larger values reflecting increased predicted binding specificity for ACE2 over ACE

**Table 2:** Top ranked fluency hits for binding to ACE2, based on a consensus ranking using the results of both models

Drug Name	Highest similarity to known binder	pBind_a	pBind_b	pBind_a_rank	pBind_b_rank	Reverse Fluency Rank (out of 20,206)	Description
Enalaprilat	0.43	7.90	8.42	4	76	17 (0.084%)	ACE inhibitor; antihypertensive drug
Orlistat	0.25	6.11	8.43	42	72	43 (0.21%)	Reversible inhibitor of lipases; obesity drug
Sotagliflozin	0.39	5.55	8.53	83	36	55 (0.27%)	Inhibits sodium–glucose co-transporters; type I diabetes drug
Tirofiban hydrochloride	0.43	8.43	8.27	1	125	34 (0.17%)	Reversible antagonist of fibrinogen binding to the GP IIb/IIIa receptor; blood thinner
Argatroban	0.54	5.99	8.40	45	83	80 (0.40%)	Inhibiting thrombin-catalyzed or induced reactions; blood thinner
Piperacillin sodium	0.54	5.51	8.44	88	67	7 (0.035%)	Binds to specific penicillin-binding proteins; antibacterial
Ramipril	0.47	6.85	8.20	17	159	3 (0.015%)	ACE inhibitor; high blood pressure
Lisinopril	0.47	7.72	8.15	7	176	16 (0.08%)	ACE inhibitor; high blood pressure
Monopril	0.47	7.84	7.98	6	240	174 (0.86%)	ACE inhibitor; high blood pressure
Captopril	0.37	7.54	7.91	9	262	5 (0.025%)	ACE inhibitor; high blood pressure
Nateglinide	0.70	7.59	7.86	8	284	69 (0.34%)	Interacts with the ATP-sensitive potassium (K + ATP) channel on pancreatic beta-cells; anti-diabetic
R-406	0.46	8.10	7.16	2	548	3735 (18.5%)	Tyrosine-protein kinase SYK inhibitor
Emricasan	0.44	7.10	8.07	14	209	250 (1.24%)	pan-caspase inhibitor

For each version of fluency run (models a and b), the predicted binding and rank is reported. A higher “pBind” signifies a higher binding affinity. A lower “Reverse Fluency” rank signifies a higher predicted specificity to the intended target

To explore other potential COVID-19 associated hits, we ran both Fluency models with TMPRSS2 as the target on the Selleckchem FDA approved drug library, and ranked hits based on performance in model a. Ombitasvir, Elbasvir, and Capecitabine are the top predicted binding hits for TMPRSS2, and Cefotiam Hexetil Hydrochloride and Bictegravir are top 10 predicted hits by both models (Table 3). Interestingly, chloroquine diphosphate was predicted by model b to bind ACE2 with a pBind of 7.8 (ranked 290 out of the FDA approved drugs for predicted binding) and TMPRSS2 with a pBind of 7.5 (ranked 210), while hydroxychloroquine sulfate was predicted by model b to bind ACE2 with a pBind of 7.9 (rank 261) and TMPRSS2 with a pBind of 7.22 (rank 307) (results not shown).

**Table 3:** Top ranked fluency hits from both models for binding to TMPRSS2

Name	Target	pBind_a_rank	pBind_a	pBind_b_rank	pBind_b
Ombitasvir	HCV Protease	1	8.06705761	22	8.22662449
Elbasvir	HCV Protease	2	7.96500254	682	6.57036352
Capecitabine	DNA/RNA Synthesis	3	7.60813904	322	7.22963095
Daclatasvir Digydrochloride	HCV Protease	4	7.49980402	86	7.91011429
Cefotiam Hexetil Hydrochloride	Others	5	7.47669792	2	8.66041851
Benzathine penicilline	Anti-infection	6	7.45924807	356	7.16500759
Betrixaban	factor Xa(fXa)	7	7.42845345	297	7.29581976
ag-120-lvosidenib	Dehydrogenase	8	7.36058712	91	7.89741182
Bictegravir	Integrase	9	7.32781744	3	8.52594948
Bivalrudin Trifluoroacetate	Thrombin	10	7.28658724	123	7.78075266
Apixaban	Factor Xa	11	7.27066994	34	8.15546131
Daclatasvir	HCV Protease	12	7.18292522	85	7.9114399
Atazanavir sulfate	HIV Protease	13	7.11152267	16	8.25314713
Edoxaban	Factor Xa	14	7.09891272	237	7.46054792
Betrixaban maleate	factor Xa?(fXa)	15	6.96175146	154	7.69741154
Nafamostat mesylate	Proteasome	16	6.9585948	166	7.64408255
Cilengitide trifluoroacetate	Integrin	17	6.95002556	696	6.55277014
Ledipasvir	HCV Protease	18	6.82781458	48	8.08298016
ARN-509	Adrenergic Receptor	19	6.76154852	76	7.95671558
Teicoplanin	Anti-infection	20	6.75152779	480	6.89225483
Vorapaxar	Protease-activated Receptor	21	6.67341661	152	7.69834948
BIBR-1048	Thrombin	22	6.66217518	87	7.9091177
Camostat Mesilate	HCV Protease	23	6.65575886	75	7.96003532
Sulbutiamine	Others	24	6.6378665	79	7.95064497
Desmopressin Acetate	V2 receptors	25	6.61611748	319	7.23214531

The “rank” column indicates the ranked position for a given model by binding prediction

**Fig. 1:**
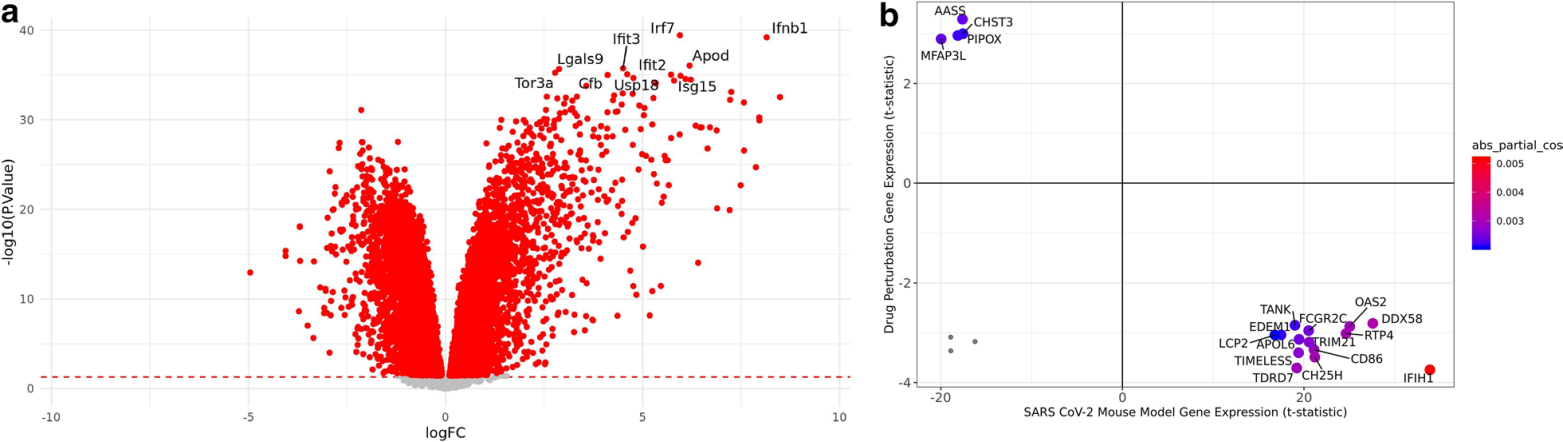
Glutamine is a top hit for cancelling out Coronavirus associated gene expression. **a** Gene expression changes associated with 2 days of MA15 infection expression in mouse pulmonary epithelial cells. X axis is log fold change, Y axis is -log10 p value. **b** Genes driving Glutamine to be a top hit. Each dot is a gene. X axis represents t-statistic of gene expression changes associated with coronavirus model, y axis represents t-statistics of gene expression changes associated with glutamine

In order to confirm or deny findings from Fluency, we applied a disease cancelling technology approach, searching for FDA approved drugs which reverse Coronavirus associated gene expression changes. Unlike Fluency, DCT was applied in a target and pathway agnostic way, capturing the full gene expression change in a data driven way. Publicly available gene expression data were downloaded from GEO (GSE68820). Healthy mice (C57BL/6NJ) were infected with MA15 (mouse version of SARS-CoV) [20]. Lung tissue was collected for gene expression analysis. A robust differential expression signal was detected between infected and uninfected mice at day 2 (Fig. 1a). Top differentially expressed genes included Irf7, Ifnb1, Apod, Ifit3, Lgals9, Tor3a, and Usp18. Multiple relevant pathways come up as significant (adj.pval < 3E−05) when performing GSEA-pre-ranked analysis, including influenza viral RNA transcription and replication, lymphocyte network, interferon signaling, Jak Stat signaling, and Graft vs Host disease. This disease signature was applied to Immuneering’s Disease Cancelling Technology to identify drugs which could potentially cancel out MA15 associated gene expression. Out of the 26,288 drugs tested, glutamine ranked 6th with a DCT cancellation score of − 0.0556337 with an adjusted *p* value < 1E−05 (Table 4). Genes changing in the opposite direction between MA15 infection and glutamine treatment are plotted in Fig. 1b. Interestingly, Glutamine is a precursor to Glutathione, which was ranked highly in Fluency results (Table 1). Thus, two orthogonal approaches (neural networks and cosine distance) used on two different data types (binding prediction and gene expression) both arrived at the same potential hit (Glutamine/Glutathione).

**Table 4:** Top reversing drugs against the MA15 (Mouse model of SARS-CoV) gene expression signature based on DCT

Drug Name	Concentration (uM)	Time (h)	DCT Score	p-value	Adjusted p-value
vitamin e	10	6	− 0.0744254	< 1.00E−05	< 1.00E−05
chembl2179387	0.04	24	− 0.0620614	< 1.00E−05	< 1.00E−05
ruxolitinib	3	24	− 0.060335	< 1.00E−05	< 1.00E−05
chembl1235119	3	24	− 0.0595284	< 1.00E−05	< 1.00E−05
cinnarizine	0.1	24	− 0.0565905	< 1.00E−05	< 1.00E−05
glutamine	1	6	− 0.0556337	< 1.00E−05	< 1.00E−05

A more negative DCT is a stronger result

## Discussion

First, we utilized an unbiased AI-based systems algorithm to interrogate 2657 FDA approved or repurposing drugs for binding to ACE2, the main SARS-CoV-2 human cell entry receptor. The rapid analysis of repurposing approved drugs for new indications allows for immediate access to potential agents that could be used for urgent emerging diseases, such as COVID-19. The ability to identify such drugs requires additional biologic validation through in vitro studies confirming receptor blockade and inhibition of SARS-CoV-2 cell entry and replication, and in vivo ideally through randomized, controlled clinical trials. During a global pandemic, however, time may not allow for usual drug development processes and repurposing of commonly available drugs may be critical. Indeed, anecdotal reports of hydroxychloroquine, azithromycin and anti-IL6 therapies have received attention [21, 22]. While hydroxychloroquine was predicted to bind to ACE2 by model b, supporting the anecdotal reports, we did not detect azithromycin or anti-IL-6 agents as these would not be anticipated to mediate therapeutic activity through ACE2 modulation. Further validation will be needed to determine if unbiased AI-based systems approaches are superior to anecdotal observations.

In the binding prediction analysis, multiple known drugs were identified as potential ACE2 inhibitors (Table 1). Not surprisingly, twelve were ACE inhibitors. This adds some confirmation that the unbiased selection accurately identified drugs with high likelihood of receptor binding. ACE inhibitors are agents commonly used for the treatment of hypertension and heart failure. This family of drugs are based on various peptide compositions and were initially selected for binding to ACE1, which catalyzes the conversion of angiotensin I to angiotensin II, thereby blocking the renin-angiotensin system (RAS), lowering systemic blood pressure, increasing sodium excretion and increased renal water output. ACE inhibitors are not known to bind to ACE2, which lacks the carboxypeptidase activity of ACE1, but does contain a zinc-binding domain, exhibits metallopeptidase activity and shares approximately 40% homology with ACE1 [23, 24]. Our model selected for preferential ACE2 binding and agents with better predicted binding values were prioritized (see Table 1). Early studies largely used angiotensin catalysis as the major readout for inhibition and whether current ACE inhibitors may block SARS-CoV-2 binding remains speculative [25]. In addition, due to the counter regulatory nature of ACE1 and ACE2 expression, it is possible that agents that downregulate ACE1 receptors may increase ACE2 receptor expression and could worsen coronavirus infection. Thus, we scanned for binding of both ACE1 and ACE2 for top hits, and ranked by predicted difference in binding. By this metric, Captopril, Enalaprilat and Monopril looked likely to inhibit both and potentially solicit this undesired feedback effect (Table 1). Ramipril is a long-acting ACE inhibitor prodrug that is converted to the active metabolite ramiprilat in the liver and may be associated with hepatic injury. Likewise, monopril is a pro-drug that undergoes transformation in the liver to the active metabolite fosinoprilat. In contrast, lisinopril is an orally active ACE inhibitor that does not undergo metabolic transformation and is excreted in the urine and does not bind to other serum proteins but may also be associated with hepatic toxicity and these drugs need to be used cautiously in patients with underlying liver disease. Captopril is a sulfhydryl-containing proline analog with potent and specific activity in blocking ACE peptidyl-dipeptidase activity. Captopril may also have anti-tumor activity through inhibition of tumor angiogenesis and promotion of anti-tumor immunity [26].

The analysis also identified a drug involved in glucose homeostasis and used in patients with diabetes mellitus as anti-hyperglycemic agents. Nateglinide (Table 1) is a derivative of phenylalanine and acts on beta-islet pancreatic cells ATP-sensitive potassium channels and stimulates insulin secretion [27]. The drug has been used for treatment of type 2 diabetes mellitus. Sotaglifozin (Table 2) is an oral inhibitor of the sodium-glucose co-transporter subtype 1 (SGLT1), expressed in the gastrointestinal (GI) tract and SGLT2, expressed in the kidneys [28]. To our knowledge, this agent have not been previously known to bind to ACE or ACE2. Glutathione is another interesting agent that was predicted by both binding AI and gene expression disease cancellation. It is an antioxidant demonstrating improved airway clearance and pulmonary function in cystic fibrosis [29]. Glutathione has also been evaluated as an adjunct in patients receiving certain chemotherapy agents following lung transplantation, and for management of HIV and Parkinson’s disease with mixed results [30].

Fostamatinib (R-406, Table 2) is an oral inhibitor of the spleen tyrosine kinase (Syk) that is converted to the active metabolite, tamatinib, and has been approved for the treatment of chronic immune thrombocytopenic purpura and is being evaluated in other autoimmune disorders, such as rheumatoid arthritis [31]. R-406 may also mediate signal transduction downstream of classical immunoreceptors, including the B-cell receptor explaining why it may be useful in treating autoimmune diseases and B cell hematologic malignancies [32]. Emricasan (Table 2), also called IDN-6556, is a thiol protease that acts as a caspase-3 inhibitor that received orphan g status by the U.S. FDA for treatment of liver disease, such as chronic hepatitis C, where it functions to protect against excessive hepatic cell apoptosis. Emricasan has been shown to decrease hepatic aminotransferases in patients with hepatitis C and other viral-induced and non-viral liver diseases [33]. The drug has also shown activity against Zika virus-mediated caspase 3 induction and blocked viral infection of neural cells in vitro [34]. The potential antiviral activity of emricasan was identified in a drug repurposing screen following the Zika virus outbreak in 2016 [34]. Fosamprenavir was identified (Table 2) and is a protease inhibitor prodrug of amprenavir, an anti-retroviral drug approved for the treatment of HIV disease. Agents with known antiviral activity against RNA viruses are especially interesting for evaluation against Coronaviruses.

Orlistat (Table 2) is a carboxyl ester and reversible inhibitor of GI lipases [35]. Orlistat was initially isolated from *Streptomyces toxytricini*, a gram-positive bacterium, and blocks hydrolysis and absorption of dietary fats and was approved in the U.S. and U.K. for the treatment of obesity. Two of the drugs identified have activity as anticoagulants, tirofiban hydrochloride (Table 1) and argatroban (Table 2). Tirofiban is a non-peptide tyrosine derivative and functions as an antagonist of the purinergic receptor, platelet glycoprotein-IIB/IIA [36]. The drug inhibits platelet aggregation and has been used for treating acute coronary syndrome and is being studied for management of ischemic stroke [37]. In contrast, argatroban is a small molecule that directly inhibits thrombin and is used for management of heparin-induced thrombocytopenia [38]. Piperacillin (Table 2) is a broad spectrum, semi-synthetic, beta-lactam, ureidopenicillin antibiotic derived from ampicillin. Piperacillin is active against gram-negative bacteria and was initially used for treating *Pseudomonas aeruginosa* infections and later as part of combination antibiotics for more complex infectious indications [39]. In contrast to macrolide antibiotics such as azithromycin which inhibit bacterial protein synthesis, piperacillin blocks bacterial wall synthesis. Since these are commonly used agents in the management of patients with pneumonia, they both merit further studies to understand their role in ACE2 modulation and potential role in management of COVID-19 infection.

To search for potential COVID-19 therapeutic approaches in an orthogonal and unbiased way, we applied our Disease Cancellation Technology to gene expression data from an animal model of SARS-CoV and ranked compounds by their ability to induce gene expression signals that counteract disease-associated signals. By this gene expression method, glutamine was a top hit for reversing Coronavirus associated changes in gene expression. Glutathione was highly ranked by Fluency for ACE2 binding and its precursor glutamine was highly ranked by gene expression DCT, suggesting both deserve further testing to explore potential benefits against SARS-CoV-2. Both glutamine and glutathione have previously demonstrated antiviral activity against herpes virus (HSV) infections [40].

## Conclusion

In summary, we used a novel AI-based systems approach to identify potential drugs currently available that are predicted to bind to ACE 2. These agents are readily available and could be rapidly assessed both in the laboratory and clinic for activity against SARS-CoV-2 infection and clinical course of COVID-19 disease. Further studies of these agents may provide new clinical strategies for patients with coronavirus diseases. Under normal circumstances, we would conduct experimental validation prior to submitting this report for publication. Given the current public health emergency, we are publishing this work now in the event that others are set up to more quickly validate, assess, and build upon these findings. Although validation is still needed, this report highlights how AI-based systems may be utilized to rapidly identify drugs for repurposing against new and emerging human diseases.

## Materials and methods

ACE2 (UNIPROT ID: Q9BYF1), ACE1 (UNIPROT ID: P12821), and TMPRSS2 (UNIPROT ID: O15393) were run separately as the protein target of Immuneering’s Fluency query. Fluency is a single universal quantitative structure–activity relationship (QSAR) deep learning model, which takes protein amino acid sequence and small molecule SMILES as input. Fluency was trained on experimental binding data from chembl 24 (model a) and chembl 25 (model b). Fluency predictions have previously been experimentally validated for multiple targets. In this case, Fluency was used to predict binding of the Selleckchem FDA approved drug library (https://www.selleckchem.com/screening/fda-approved-drug-library.html) separately to ACE2, ACE1, and TMPRSS2. For top hits, fluency was run in reverse (predicting binding of a single small molecule to 20,206 human proteins) to score specificity. Predicted binding scores for ACE1 and ACE2 were compared for top hits to assess predicted specificity for ACE2 over ACE1 in each model (as reflected in the “pBind_x_ACE2-pBind_x_ACE” columns). Similarity to known binders (reported pChEMBL value greater than 7 in the ChEMBL database) to ACE2 was computed using Tanimoto distance of molecular fingerprints from RDKit in Python. Top ranked Fluency hits were filtered by evaluating individual rankings from model a and model b, as well as the average rank of predictions and the combined pBIND scores of both models.

Gene expression data was downloaded from GEO (GSE68820). The processed data which was background corrected, quantile normalized, and summarized after outlier removal by the author was used [20]. For each of the time points, differential expression was calculated between the MA15 (SARS-CoV) virus infected wild type mice lung samples and the mock-inoculated wild type mice using the limma R-package version 3.40.6 [41]. Immuneering leveraged its previously described [42] and validated [43, 44] DCT, and ran the SARS-CoV disease signature against the LINCS drug perturbation database [45]. Results were filtered for adjusted p-value significance and maximal disease cancellation score.

## Data Availability

Publicly available data was analyzed for this manuscript

## Abbreviations

ACE1: Angiotensin converting enzyme 1
ACE2: Angiotensin converting enzyme 2
AI: Artificial Intelligence
APOD: Apolipoprotein D
COVID-19: Coronavirus disease-2019
DCT: Disease Cancelling Technology
ER: Endoplasmic reticulum
FDA: Food & Drug Administration
GEO: Gene Expression Omnibus
GI: Gastrointestinal
HSV: Herpes virus
IFIT3: Interferon Induced Protein With Tetratricopeptide Repeats 3
IFNB1: Interferon Beta 1
IL6: Interleukin 6
IRF7: Interferon Regulatory Factor 7
LGALS9: Galectin 9
MA15: Mouse Adopted SARS Virus
MERS: Middle East Respiratory Syndrome
pBind: Probability of Binding (metric from Fluency, higher number = higher predicted binding)
QSAR: Quantitative structure–activity relationship
R&D: Research & Development
RAS: Renin-angiotensin system
SARS: Severe acute respiratory syndrome
SARS-CoV-2: Severe acute respiratory syndrome coronavirus 2
SGLT1: Sodium-glucose co-transporter subtype 1
SMILES: Simplified Molecular Input Line Entry System
SYK: Spleen tyrosine kinase
TMPRSS2: Transmembrane Serine Protease 2
TOR3A: Torsin Family 3 Member A
TRS: Transcriptional regulatory sequence
UTR: Untranslated Region
Usp18: Ubiquitin Specific Peptidase 18
